# Author Reflections: Retroperitoneal and Extremity Liposarcoma: An Opportunity to Harmonize Heterogeneity

**DOI:** 10.1245/s10434-026-19888-4

**Published:** 2026-05-29

**Authors:** William W. Tseng, Alessandro Gronchi

**Affiliations:** 1 Division of Surgical Oncology, Department of Surgery, City of Hope National Medical Center, Duarte, CA USA; 2 Sarcoma Service, Department of Surgery, Fondazione IRCCS Istituto Nazionale dei Tumori, Milan, Italy

## Past

Retroperitoneal liposarcomas (RP LPS) are typically large tumors for which surgery can be very challenging due to potential involvement of organs and critical structures. Even after complete resection, which at some centers may involve a team of surgeons (e.g., Surgical Oncology, Urology, Vascular Surgery), locoregional recurrence remains a significant issue for this type of sarcoma.^1^ In fact, for many patients with RP LPS, the ability to achieve cure is quite elusive. Histologic subtype (well-differentiated [WD] vs dedifferentiated [DD]) and grade are recognized as factors that clearly impact outcome and also may help guide utilization of radiation therapy (STRASS/STREXIT data^2^) and potentially, chemotherapy (pending STRASS 2 data). Although no other clinicopathologic factor or molecular marker has been identified to date with more definitive clinical impact, heterogeneity within subtype (WD, DD) is well-recognized among sarcoma specialists. Some WD tumors are lipoma-like clinically and histologically. In contrast, there are extremely rare variants of DD (e.g., rhabdomyoblastic differentiation) reported to metastasize and lead to patient mortality for more than 80% of patients within 1 year.^3^

## Present

In the current study, we hypothesized that disease heterogeneity also can encompass anatomic location within the RP, determined by careful review of preoperative imaging and with intraoperative assessment. Our findings define a subset of primary WD RP LPS confined to or arising from the psoas muscle for which surgery alone appears to be curative with a median follow-up period of 8.8 years.^4^ The clinical outcomes approximate those of WD disease in the extremity. Moreover, the resections performed in these RP psoas tumors were much more conservative than those for patients with “conventional” RP non-psoas tumors, who undergo multiorgan resection nearly three times more frequently. With further validation of the study findings, treatment for WD LPS of the psoas muscle can potentially be modified to spare these patients from extensive organ resection, radiation, or chemotherapy when clinically appropriate.

## Future

The findings of the current study raise the broader logistical need to harmonize efforts between colleagues who treat the same tumor type. In LPS, among surgeons, this includes Surgical Oncology and Orthopedics (*). Harmonization of efforts should include both the approach to clinical management and research endeavors. Is a histologically bland MDM2-amplified tumor of the RP (labeled “WD liposarcoma” by Surgical Oncology) truly the same as a tumor of the extremity (labeled “atypical lipomatous tumor” by Orthopedic Oncology)? Although earlier studies have shed some light,^5^ we currently are better poised to revisit this topic with expanded, collaborative experience and new research tools. As the field continues to recognize disease heterogeneity (e.g., through deep molecular characterization) by harmonizing across anatomic sites, together we have the real potential to uncover novel and much needed insights into the disease itself!

## References

[1] Sun S, Cardona K, Tseng WW. Surgical management of retroperitoneal liposarcoma: opportunities for multimodality treatment, including systemic therapy. *Cancer Med*. 2025;14:e71129. 10.1002/cam4.71129. 40778536 10.1002/cam4.71129 PMC12332537

[2] Callegaro D, Raut CP, Ajayi T, et al. Preoperative radiotherapy in patients with primary retroperitoneal sarcoma: EORTC-62092 trial (STRASS) versus off-trial (STREXIT) results. *Ann Surg*. 2023;278:127–34. 10.1097/SLA.0000000000005492. 35833413 10.1097/SLA.0000000000005492

[3] Gronchi A, Collini P, Miceli R, et al. Myogenic differentiation and histologic grading are major prognostic determinants in retroperitoneal liposarcoma. *Am J Surg Pathol*. 2015;39:383–93. 10.1097/PAS.0000000000000366. 25581729 10.1097/PAS.0000000000000366

[4] Tseng WW, Barretta F, Fiore M, et al. Well-differentiated liposarcoma of the psoas muscle: a distinct anatomic subset with highly favorable outcomes. *Ann Surg Oncol*. 2026. 10.1245/s10434-026-19738-3. 42086973 10.1245/s10434-026-19738-3 PMC13337796

[5] Dalal KM, Kattan MW, Antonescu CR, Brennan MF, Singer S. Subtype-specific prognostic nomogram for patients with primary liposarcoma of the retroperitoneum, extremity, or trunk. *Ann Surg*. 2006;244:381–91. 10.1097/01.sla.0000234795.98607.00. 16926564 10.1097/01.sla.0000234795.98607.00 PMC1856537

